# Evaluating the ability of citizen scientists to identify bumblebee (*Bombus*) species

**DOI:** 10.1371/journal.pone.0218614

**Published:** 2019-06-24

**Authors:** Steven Falk, Gemma Foster, Richard Comont, Judith Conroy, Helen Bostock, Andrew Salisbury, Dave Kilbey, James Bennett, Barbara Smith

**Affiliations:** 1 The Centre for Agroecology, Water and Resilience, Coventry University, Coventry, United Kingdom; 2 The Bumblebee Conservation Trust, Eastleigh, United Kingdom; 3 The Royal Horticultural Society, Wisley, United Kingdom; 4 Natural Apptitude, Bristol, United Kingdom; National Taiwan Normal University, TAIWAN

## Abstract

Citizen science is an increasingly popular way of engaging volunteers in the collection of scientific data. Despite this, data quality remains a concern and there is little published evidence about the accuracy of records generated by citizen scientists. Here we compare data generated by two British citizen science projects, Blooms for Bees and BeeWatch, to determine the ability of volunteer recorders to identify bumblebee (*Bombus*) species. We assessed recorders’ identification ability in two ways–as recorder accuracy (the proportion of expert-verified records correctly identified by recorders) and recorder success (the proportion of recorder-submitted identifications confirmed correct by verifiers). Recorder identification ability was low (<50% accuracy; <60% success), despite access to project specific bumblebee identification materials. Identification ability varied significantly depending on bumblebee species, with recorders most able to correctly identify species with distinct appearances. Blooms for Bees recorders (largely recruited from the gardening community) were markedly less able to identify bumblebees than BeeWatch recorders (largely individuals with a more specific interest in bumblebees). Within both projects, recorders demonstrated an improvement in identification ability over time. Here we demonstrate and quantify the essential role of expert verification within citizen science projects, and highlight where resources could be strengthened to improve recorder ability.

## Introduction

Citizen science is an increasingly popular tool for engaging volunteers in the collection of scientific data, especially within the field of ecology [1]. In recent years there has been a rapid increase in the number, size and scope of citizen science initiatives, particularly in Europe and North America [2,3]. This is partly a result of rapid technological developments including online recording, project apps and digital photography, which have facilitated improved data verification and validation [4–7]. One of the main strengths of citizen science is that it allows researchers to collect data across broad geographic scales and in private spaces such as gardens, both of which are difficult and expensive to achieve using traditional field research [8]. Citizen science projects can also contribute discussion as well as data [9] and can have positive impacts on participants’ scientific skills, knowledge, attitudes and behaviour [6,10–12].

Although citizen science projects are increasingly recognized as valuable sources of data, the quality of data submitted by non-specialists remains a concern [7,13]. Citizen science data is frequently perceived as low quality and unreliable [3], but the potential for error and bias is poorly understood [8]. Some studies have found that the quality of data collected by volunteers can be more variable than that collected by professionals [14,15], while others have found it comparable [16–18]. Within ecology, most citizen science projects have attempted to address data quality issues with verification and validation of data by experts (‘verifiers’) [7,19,20].

There is a long history of biological recording in Britain and Ireland, traditionally comprising self-supporting networks of volunteer naturalists with high levels of expertise, compiling sightings data and known as ‘recording schemes’. The majority of these have been based around the collection of ad hoc observation records (e.g. the UK Ladybird Survey www.ladybird-survey.org, or Hoverfly Recording Scheme www.hoverfly.org.uk), but others have been more standardised (e.g. the UK Butterfly Monitoring Scheme [21]). In recent years the growth of digital technology, principally the internet, digital photography and smartphones, has led to increasing interest in engaging non-expert citizen scientists in biological recording. Widespread concerns about pollinating insect declines [22–24] have brought the monitoring of pollinating insects to the forefront of this movement, with popular projects including the Friends of the Earth's Great British Bee Count (https://friendsoftheearth.uk/bee-count) in Great Britain and Bumble Bee Watch (www.bumblebeewatch.org) in North America.

Due to the difficulties of species-level identification, coupled with the targeting of non-specialist recorders, many new citizen science projects group insects into broad categories or species groups [25,26]. This has particularly been the case for pollinator monitoring projects (e.g. the Flower Insect-Timed Counts used for the national Pollinator Monitoring Project, PoMS: www.ceh.ac.uk/our-science/projects/pollinator-monitoring, or the Great British Bee Count). Others, such as BeeWatch (https://beewatch.abdn.ac.uk/) or the Bees, Wasps and Ants Recording Society (BWARS) (http://www.bwars.com) have sought to maintain accurate species-level recording through expert verification, largely of photographs submitted with the records, and an acceptance that a higher proportion of records will be rejected as unidentifiable compared to the broad-group approach.

There is little published evidence about the quality and accuracy of citizen science data [12,19,20,27]. This is especially true of insect recording schemes, particularly at the species level. Although much of the knowledge about data quality and accuracy does exist, it typically only lies with the expert verifiers for each recording scheme [5]. Developing a better understanding of the strengths, limitations and biases of citizen science data in pollinator monitoring projects is important because it is growing in popularity throughout the world, and because implementing a sustainable long-term monitoring programme is one of the aspirations of ‘The National pollinator strategy: for bees and other pollinators in England’ [28].

In 2016, the three-year Blooms for Bees project was launched to gather data on the floral preferences of bumblebee species in British gardens and allotments, thus complementing other bee surveys by introducing a greater focus on horticulture and flower visitation. The project was designed to use smartphone technology, and a free project app featuring a bumblebee identification guide was created. The app allowed participants to submit records including photographic evidence which enabled expert verification of bumblebees to species level. Bumblebees are members of the genus *Bombus* and are large, brightly coloured insects that are easily observed by non-specialists. Most species are striped black and yellow, and the external differences between species is often (though not always) clear. There are a small number of common species and several of these have colour patterns that are unique, or shared only by rare congeners, making several species very distinctive, and thus suitable for a citizen science project.

In this paper, the insect data from the first year of the Blooms for Bees project is assessed and compared to that collected by the BeeWatch project, run by Aberdeen University and the Bumblebee Conservation Trust, to explore for the first time the ability of citizen scientists to identify bumblebees to species level. We assessed recorders’ identification ability in two ways–as recorder accuracy (the proportion of expert-verified records correctly identified by recorders) and recorder success (the proportion of recorder-submitted identifications confirmed correct by verifiers). So for example, in Blooms for Bees, recorders submitted a total of 474 records as *B*. *hortorum*. Expert verification confirmed that 23 of these records were *B*. *hortorum*, and identified a further 47 records of *B*. *hortorum* from the rest of the dataset (as some records that were submitted as other species were in fact *B*. *hortorum*), producing a total of 70 records of *B*. *hortorum* across the whole dataset. In this case, recorder accuracy was therefore 33% (23 of 70 records), and recorder success was 5% (23 of 474 records).

We hypothesized that recorder identification accuracy and success rates would 1) vary according to bumblebee species, 2) be lower in the Blooms for Bees project than in the BeeWatch project because of project audience (the gardening community vs largely individuals with a specific interest in bumblebees) and 3) improve over time as a result of verification feedback and learning/self-correction.

## Materials and methods

### Blooms for Bees project

Blooms for Bees was developed by Coventry University’s Centre for Agroecology, Water and Resilience (CAWR), in partnership with the Royal Horticultural Society (RHS), Bumblebee Conservation Trust (BBCT) and Garden Organic. The project app was developed by Natural Apptitude (www.natural-apptitude.co.uk) and launched in April 2017.

The project audience was home gardeners, who were recruited using the websites, newsletters and social media activity of the project and the project partners. Participants were asked to choose any plant with at least one open flower from their garden or allotment, and photograph the visiting bumblebees during the five-minute survey period (S1 Protocol). Data and photographs were submitted through the app, which also included a bumblebee identification guide. Each survey record included the date, time, the location (accurate to a horizontal error of approximately 8m [29]), a photograph of the survey plant, the name of the plant, the number of open floral units, and a photograph and provisional species identification for each bumblebee seen. Floral units were defined as single simple flowers, or one capitulum, umbel or flower spike. Recorders were encouraged to submit as many surveys as they wished. All bumblebee records received were verified/corrected by experts on the project team, and feedback sent to the recorder.

Records submitted by 485 citizen scientists between 2 April 2017 and 5 November 2017 (n = 4,200) were used for this analysis. This period represents the first year of data collection, and ran from the time the app was launched until bumblebees were largely inactive because of the cold weather.

### BeeWatch project

BeeWatch was developed by the University of Aberdeen in partnership with the Bumblebee Conservation Trust (BBCT), and the digital portal was launched in August 2011. The initial project audience was members of the BBCT, but the user base has grown beyond this over time. Like Blooms for Bees, BeeWatch relies on citizen scientist participants to submit photographs of bumblebees, but collects ad hoc records rather than using a timed survey approach. Records are submitted via an online interface and consist of a bumblebee photograph, provisional species identification, location and date of the sighting. Recorders are encouraged to use the BeeWatch website resources, including a simple binomial key, to identify their bumblebee. All records are verified/corrected by experts at the University of Aberdeen or BBCT and automated feedback is provided to the recorder [30–32].

Records submitted by 3,427 citizen scientists between September 2011 and September 2015 (n = 11,509) were used for this analysis.

### Expert verification

Verification ensures the accuracy of the species identification [33,34]. In both projects, verification involved inspecting the photograph associated with each bumblebee record received, and either confirming or correcting the recorder-submitted species identification. In Blooms for Bees, expert verification was carried out by Steven Falk (SF). Within BeeWatch, several people verified records over the survey period: during 2013–15 this was primarily Richard Comont (RC), and all records of rare or scarce species submitted during 2011–13 (those listed as priority species by the Joint Nature Conservation Committee [35]), as well as a sample of the common species, were re-checked at this time.

Both projects provided recorders with ‘remote training’ in the form of the correct identity of their record and also additional feedback. In Blooms for Bees, this consisted of any comments that the verifier regarded as helpful, usually within two to three weeks of the submission. In BeeWatch this involved automated natural language generated feedback within four weeks of submission [30–32].

In the Blooms for Bees project, during one hour, approximately 35 records could be verified and feedback written and sent to the recorder by SF. Verification speed was higher in the BeeWatch project, as a result of higher quality photographs (as recorders tended to use digital cameras rather than smartphones) and automated natural language generated feedback (to explain differences between the recorder-submitted species and the expert-verified species), with RC achieving up to 240 verifications per hour.

### Data analysis

We assessed the recorders’ bumblebee identification ability in two ways: recorder accuracy and recorder success. Recorder accuracy was defined as the proportion of expert-verified records correctly identified by recorders, while recorder success was taken as the proportion of provisional recorder-submitted species identifications that were confirmed correct by verifiers. Recorder success is dependent on the quality of the submitted photograph, as well as the recorder’s identification ability. It is particularly important as it is the main way that verifiers and recording scheme organisers can assess the ability of unknown recorders. For instance, it is implemented in the Biological Records Centre’s iRecord system [36].

For both datasets, we removed the records that could not be identified to species level. Poor-quality photographs, or those that do not show the salient features, make species identification difficult or impossible, even for experts [37], and the records that experts could not confidently assign to a species were not included in the analysis. We then compared the recorder-submitted bumblebee identification to the expert’s determination. Where these differed, the expert was assumed to be correct and the recorder’s submitted identification was marked as wrong.

In the statistical language R, version R 3.5.1 [38] we used Generalised Linear Models with a binomial or quasibinomial error distribution (as appropriate) and a logit link function to test for differences between the proportion of records correctly identified across months, years, species, and between the two datasets. This was carried out for both the proportion of the expert-verified sightings of each species which were correctly identified by recorders, and for the proportion of provisional recorder-submitted identifications that were confirmed correct by verifiers. To avoid biases in recorder accuracy and success introduced by recorders submitting multiple records and potentially improving their identification ability over time, we only used the first record submitted by each recorder in the two projects.

Taking an information theoretical approach, we used Akaike’s Information Criterion (AIC) to select the model with the best fit to the data in each case [39,40]. Where models were determined to be over-dispersed, we calculated quasi-AIC (QAIC), adjusting for over-dispersion in quasi-error structures by dividing the residual deviance (-2 log likelihood) with the over-dispersion parameter of the most complex model as the sum of squares Pearson’s residuals divided by the number of degrees of freedom [41]. Adding extra explanatory variables increased the complexity of the model, so unless the addition reduced QAIC by at least two the extra variable was deemed to have not sufficiently improved the model fit to be worth retaining in the model. Models with the lowest QAIC (bearing in mind the previous caveat) were considered to be the models with the best compromise between bias and variance. These ‘best models’ (model 1 in Tables 1 and 2) were then used throughout. We also performed an ANOVA with F or Chi-squared test (as appropriate for quasibinomial or binomial models) on model variables to determine the relative importance of individual variables once the ‘best’ models had been determined using the information theoretic approach.

**Table 1:** Fit of different models for recorder accuracy, considering only each recorder’s first submitted record (so with no improvement over time).

Model number	Model terms	Residual deviance	Residual df	QAIC	ΔQAIC
1	Dataset + Species + Year + Month	3883.1	3481	4612.213	-
2	Dataset + Species + Year	3912.9	3492	4625.042	12.829
3	Dataset + Species + Month	3925.1	3482	4652.419	40.206
4	Month + Species	3927.2	3483	4653.868	41.655
5	Dataset + Species	3942.3	3493	4657.406	45.193
6	Dataset + Month	4447.7	3504	5222.249	610.036

**Table 2:** Fit of different models for recorder success, considering only each recorder’s first submitted record (so with no improvement over time).

Model number	Model terms	Residual deviance	Residual df	QAIC	ΔQAIC
1	Dataset + Species + Year	2152.9	2295	2225.386	-
2	Dataset + Species + Year + Month	2130.7	2284	2224.975	-0.411
3	Dataset + Species + Month	2143.3	2285	2235.661	10.275
4	Dataset + Species	2168.0	2296	2238.605	13.219
5	Month + Species	2217.1	2286	2308.262	82.876
6	Dataset + Month	3037.8	2306	3097.503	872.117

To examine any change in recorders’ identification ability over time we used Generalised Linear Models with a quasibinomial error distribution and logit link function to regress each identification’s status (correct/wrong) with the recorders’ experience at the time with the project (taken as the number of surveys they had completed at that date). This was carried out for recorders who submitted more than two records, and also for recorders who submitted more than 10 records, as improvement cannot be demonstrated with a single record.

## Results

Blooms for Bees received 4,200 bumblebee records. Following expert verification, 3,011 records (72%) could be assigned to a precise bumblebee species or the *B*. *lucorum/terrestris* aggregate taxa. A total of 833 records (20%) could not be verified to species level due to lack of, or poor quality, photographic evidence. A total of 356 records (8%) were of non-bumblebee species. Records submitted to the Blooms for Bees project claimed sightings of 23 bumblebee species, and verification reduced this number to 15 confirmed species, plus the *B*. *lucorum/terrestris* aggregate taxa.

BeeWatch received 11,509 bumblebee records between September 2011 and September 2015. Following expert verification, 9410 records (82%) were assigned to a bumblebee species or the *B*. *lucorum/terrestris* aggregate taxa. A total of 1280 bumblebee records (11%) could not be verified with certainty and 819 records (7%) were of non-bumblebee species. Records claimed sightings of 22 bumblebee species, and verification confirmed 22 species, plus the *B*. *lucorum/terrestris* aggregate taxa (which was not a submittable option in this project).

The *B*. *lucorum* complex consists of three cryptic species which are impossible to separate without DNA analysis [42]: *B*. *lucorum*, *B*. *magnus*, and *B*. *cryptarum*. In both projects, the three species were grouped together as *B*. *lucorum sensu lato*, as there is a chance that some of the *B*. *lucorum* and *B*. *lucorum/terrestris* submissions represent *B*. *magnus* and *B*. *cryptarum*. DNA analysis from specimens would be required to confirm this and this was beyond the scope of these projects.

### Recorder identification ability by species

**Fig 1:**
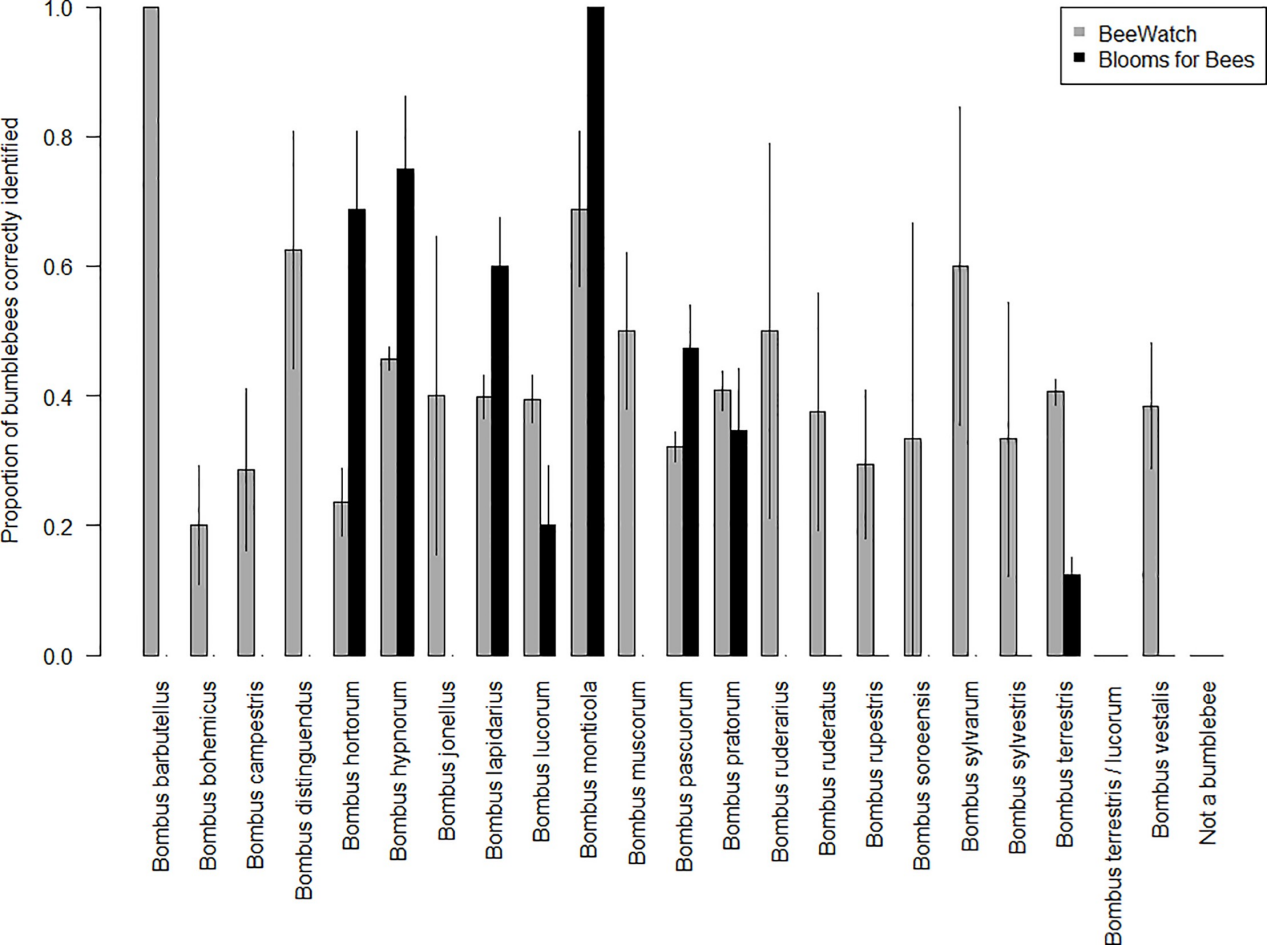
Recorder accuracy by bumblebee species, considering only each recorder’s first submitted record. Bars indicate the proportion of the expert-verified records which were correctly identified by recorders per bumblebee species in each project. ‘Not identifiable’ records (i.e. those which could not be confirmed from the photographs supplied) may have contained some correct records. Both projects had the option to not specify a species, but both were aimed at recording bumblebees and neither had an option to record sightings as members of any other group. Error bars show ± SE.

Recorder accuracy (the proportion of expert-verified records correctly identified by recorders) was found to vary significantly between bumblebee species (F_22,3493_ = 28.02, p<0.001 for model 1 in Table 1, Fig 1). The highest overall accuracy rating in Blooms for Bees, for a species with more than 10 records, was 68% for *B*. *hypnorum* (68 correctly identified from the expert-verified n = 100), and the equivalent species in the BeeWatch dataset was *B*. *distinguendus*, identified correctly 78% of the time (25 correctly identified from the expertly-verified n = 32) (S1 Table). The only species which was accurately identified 100% of the time was *B*. *monticola* in Blooms for Bees, albeit for just two records submitted by one recorder (S1 Table).

**Fig 2:**
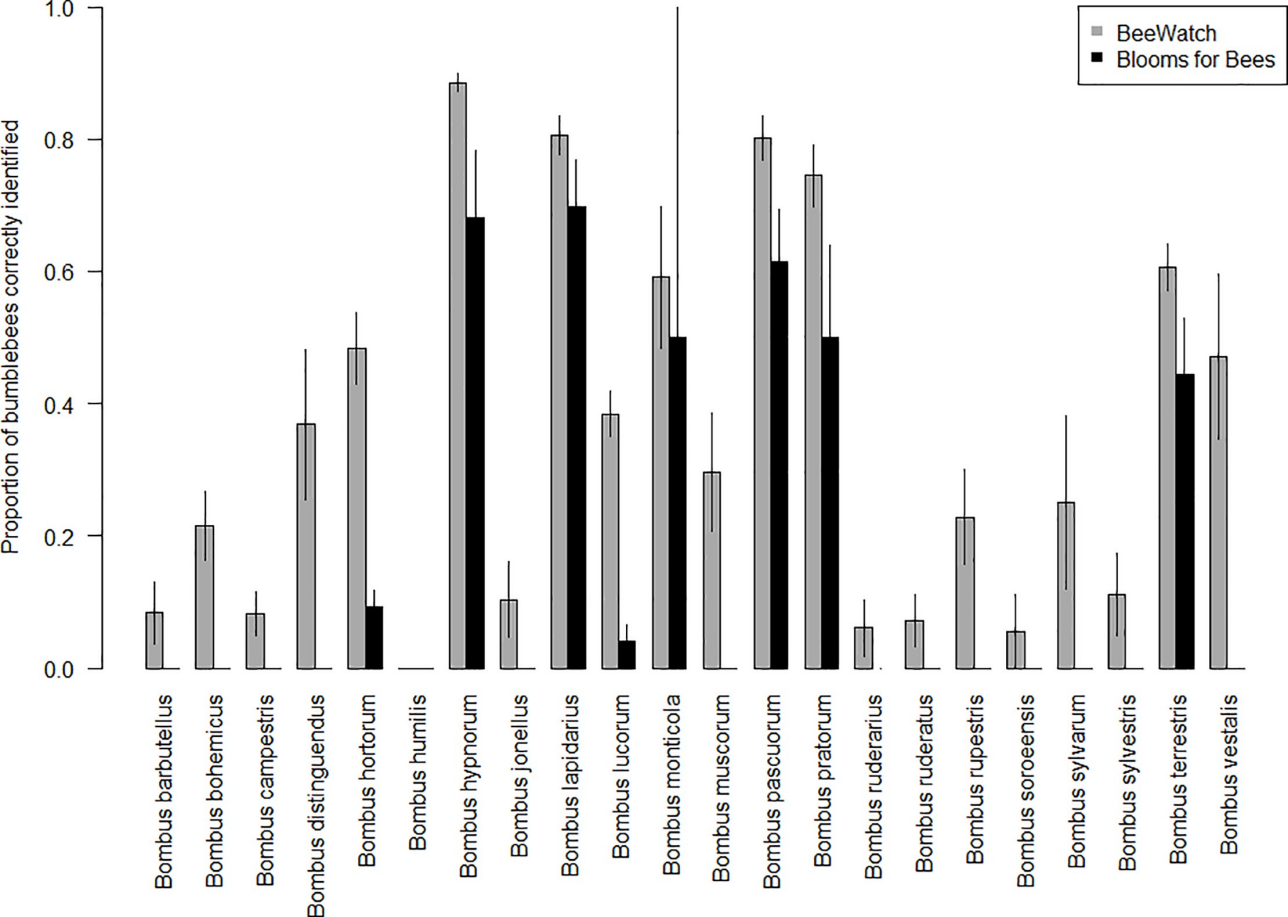
Recorder success by bumblebee species, considering only each recorder’s first submitted record. Bars indicate the overall proportion of recorder-submitted identifications which were confirmed correct by verifiers per bumblebee species in each project. ‘Not identifiable’ records (i.e. those which could not be confirmed from the photographs supplied) may have contained some correct records. Both projects had the option to not specify a species, but both were aimed at recording bumblebees and neither had an option to record sightings as members of any other group. Error bars show ±SE.

Recorder success (the proportion of recorder-submitted identifications which were confirmed correct by verifiers) also varied significantly between species (Species: χ^2^ = 911.17, p<0.001 for model 1 in Table 2, Fig 2). In Blooms for Bees, the best-identified species overall were *B*. *pascuorum* (72% of 651 submitted records were correct), *B*. *lapidarius* (70% of 279 records were correct), and *B*. *pratorum* (61% of 225 records were correct), with all other species correctly identified on less than 60% of occasions (S1 Table). Recorders in BeeWatch were best able to correctly identify *B*. *hypnorum* (87% of 961 submitted records were correct), *B*. *pascuorum* (84% of 796 records were correct), and *B*. *lapidarius* (83% of 663 records were correct), with all other species correctly identified on less than 80% of occasions (S1 Table).

Recorder success was very low (<20%) for six species confirmed in the Blooms for Bees dataset and four species confirmed in the BeeWatch dataset (Table 3). Several species were never correctly identified by Blooms for Bees recorders, namely *B*. *jonellus*, *B*. *muscorum* and *B*. *ruderatus*, although these were present in low numbers (S1 Table). The lowest identification success rates for a species with more than 10 verified records in the Blooms for Bees dataset was *B*. *vestalis*, followed by *B*. *sylvestris*. The least correctly-identified species in BeeWatch were *B*. *humilis*, *B*. *ruderarius*, *B*. *ruderatus* and *B*. *soroeensis*. It should be noted that these species are very difficult to confirm from photographs, so some sightings may have been correct, but with insufficient supporting evidence to allow them to be confirmed.

**Table 3:** Bumblebee species with overall recorder accuracy rates of less than 20%. Only species that were confirmed in the two datasets are presented.

Project and species	Number of records submitted by recorders	Number of records confirmed following expert verification	Number of records correctly identified by recorders	Recorder accuracy overall (%)	Recorder success overall (%)
**BeeWatch**					
*Bombus humilis*	98	9	4	44	4
*Bombus ruderarius*	114	10	6	60	5
*Bombus ruderatus*	126	14	7	50	6
*Bombus soroeensis*	133	25	17	68	13
**Blooms for Bees**					
*Bombus jonellus*	36	2	0	0	0
*Bombus muscorum*	11	1	0	0	0
*Bombus ruderatus*	8	3	0	0	0
*Bombus hortorum*	474	70	23	33	5
*Bombus vestalis*	13	33	2	6	15
*Bombus sylvestris*	16	13	3	23	19

### Recorder identification ability by project

**Fig 3:**
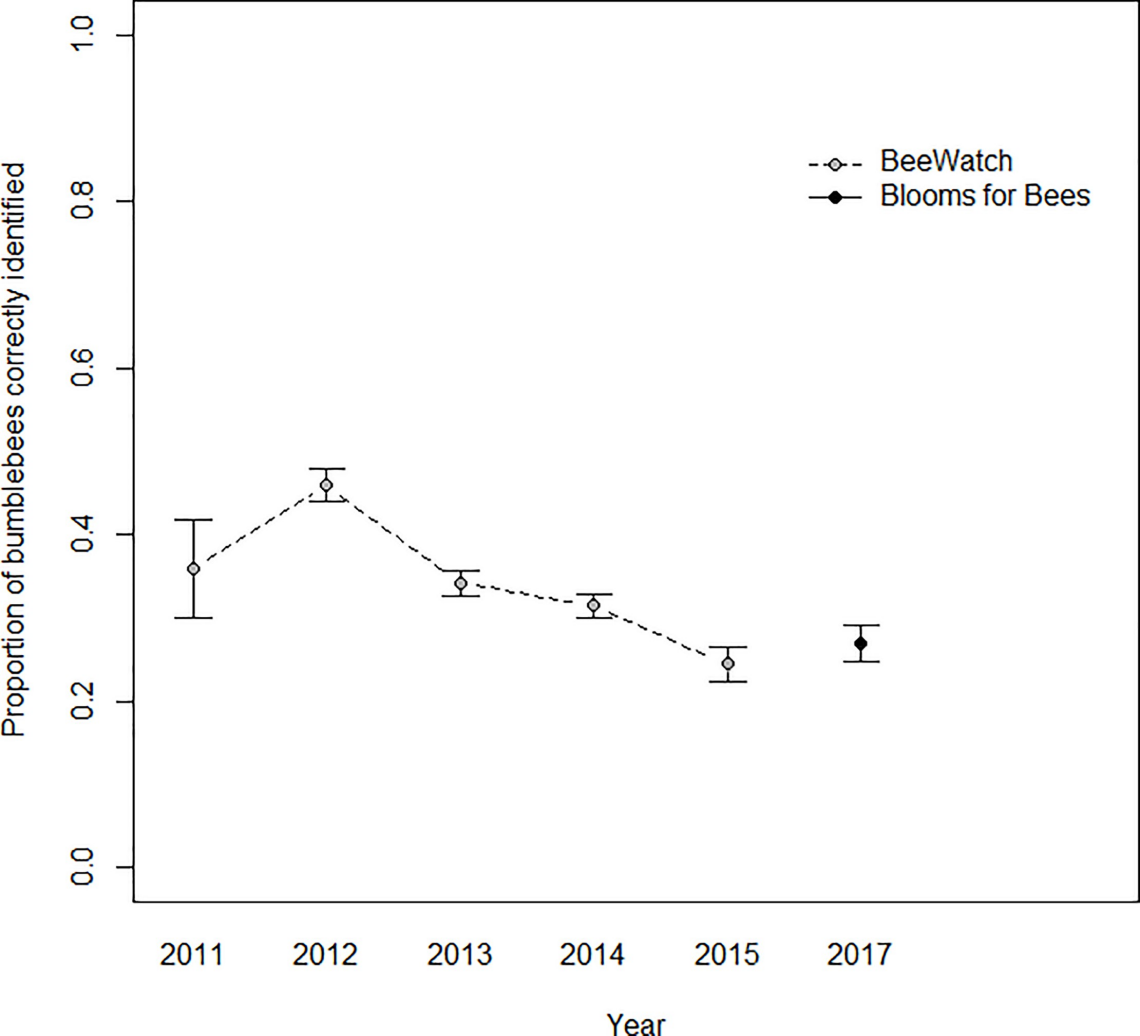
Recorder accuracy by year, considering only each recorder’s first submitted record. Points indicate the mean proportion of expert-verified sightings which were correctly identified by recorders per year and project. Error bars show ±SE.

Recorder accuracy (the proportion of expert-verified records correctly identified by recorders) varied significantly between the two project datasets, with BeeWatch recorders having significantly greater ability to correctly identify bumblebee species than Blooms for Bees recorders (Dataset: F_1,3515_ = 9.88, p = 0.0017, Fig 3). Overall, of the records that could be verified to species level, in BeeWatch 49% (4580 of 9410 records) were correctly identified by the recorder who submitted them, whereas in Blooms for Bees 44% (1,322 of the 3,011 records) were correctly identified by the recorder who submitted them.

**Fig 4:**
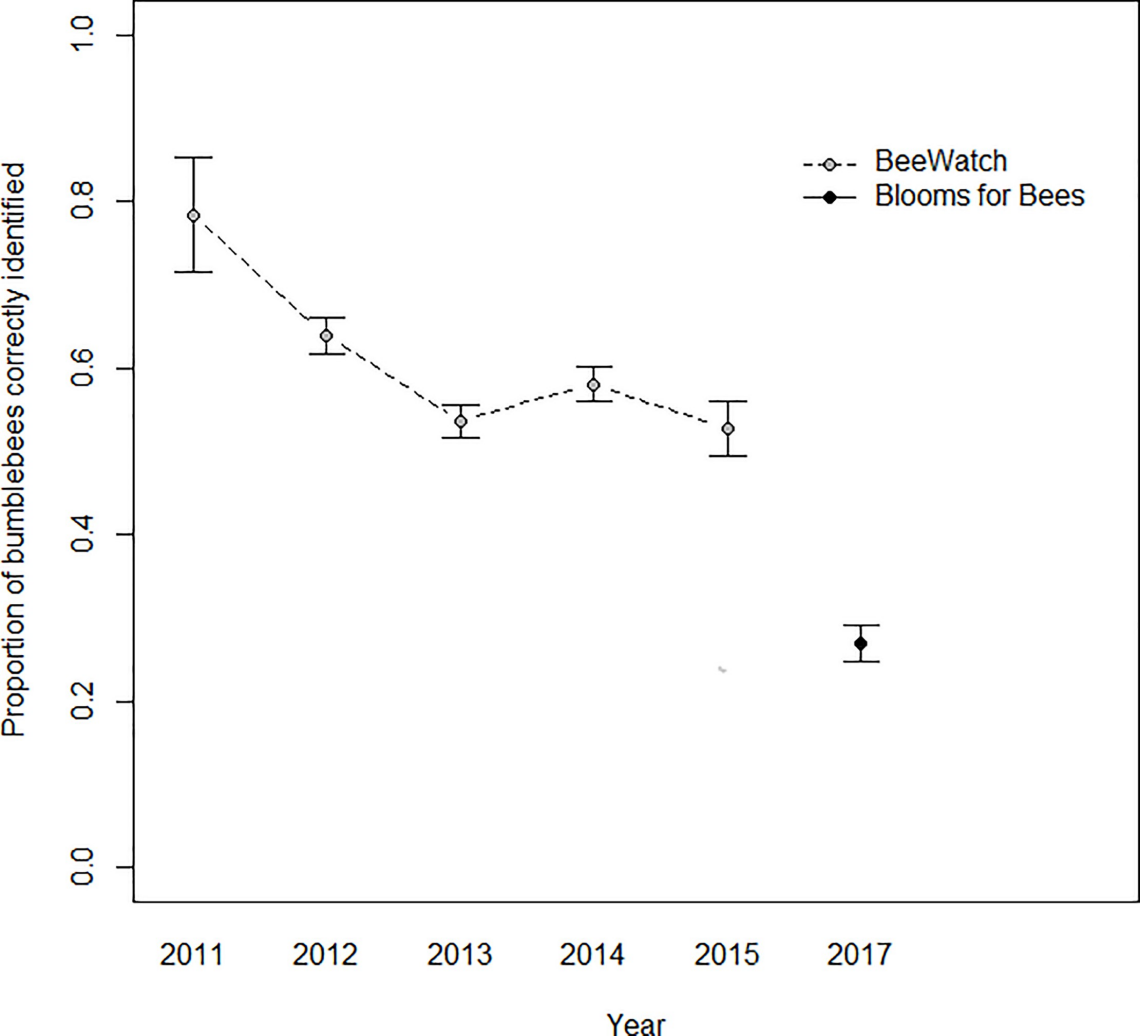
Recorder success by year, considering only each recorder’s first submitted record. Points indicate the mean proportion of recorder-submitted identifications which were confirmed by expert verifiers per year and project. Error bars show ±SE.

Mean recorder success (the proportion of recorder-submitted identifications which were confirmed correct by verifiers) also varied significantly between the two projects (Dataset χ^2^_1,2317_ = 13.54, p<0.001, Fig 4). Again, BeeWatch recorders were significantly more likely to correctly identify their bumblebees. Of the overall records that were submitted with provisional species identifications, 59% were correct in BeeWatch (4,580 of 7,699 records), whereas only 40% were correct in Blooms for Bees (1,322 of 3,342 records). This discrepancy may in part be explained by different recording behaviours, with Blooms for Bees recorders being more likely to attempt an identification than BeeWatch recorders. In Blooms for Bees, just 7% of records (282 of 4,200 records) were submitted as ‘unknown species’, whereas in BeeWatch this proportion was much higher at 33% (3,810 of 11,509 records).

### Recorder identification ability over time

**Fig 5:**
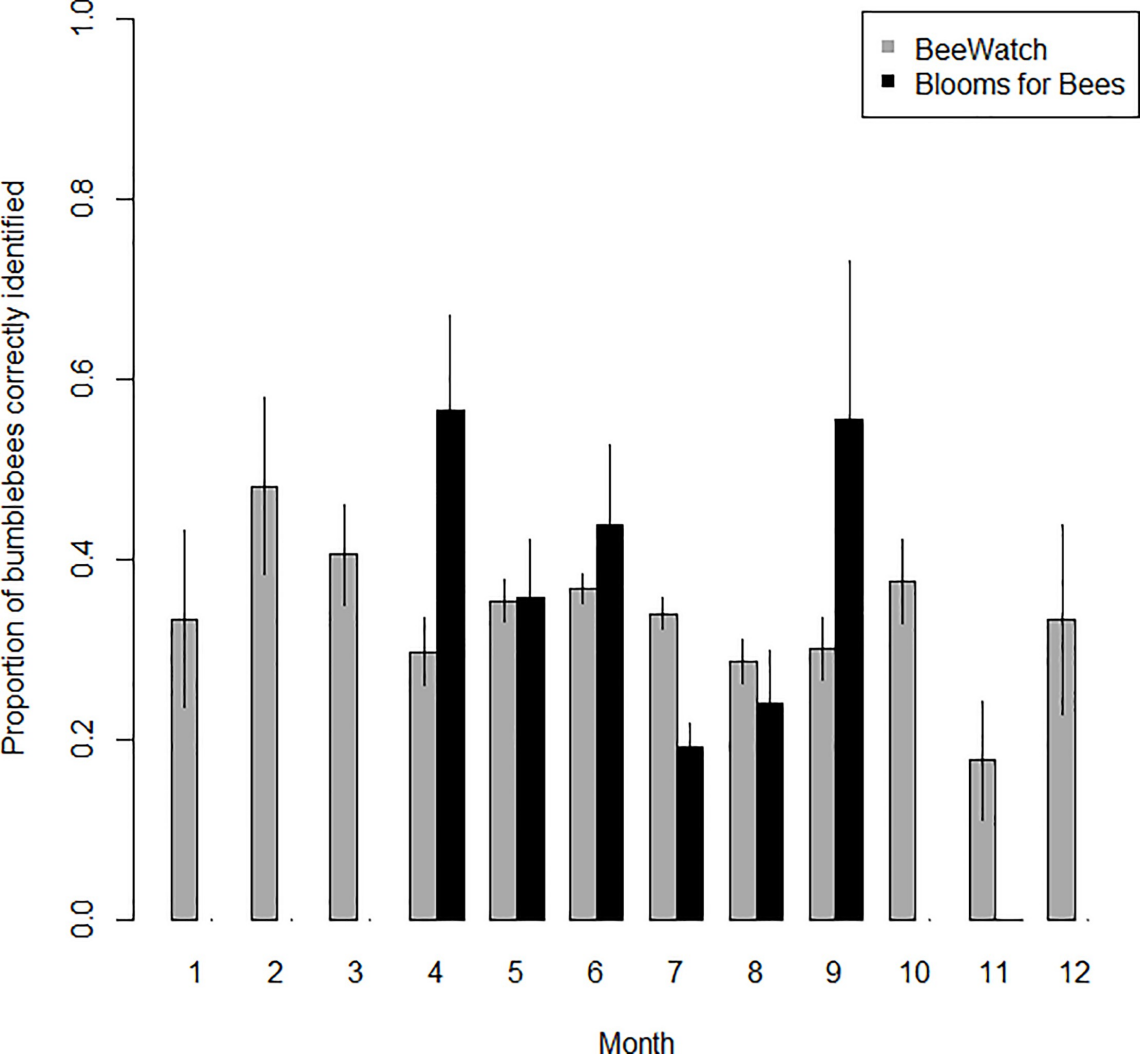
Recorder accuracy by month, considering only each recorder’s first submitted record. Bars indicate the proportion of expert-verified bumblebee sightings which were correctly identified by recorders per month and project. Error bars show ±SE.

**Fig 6:**
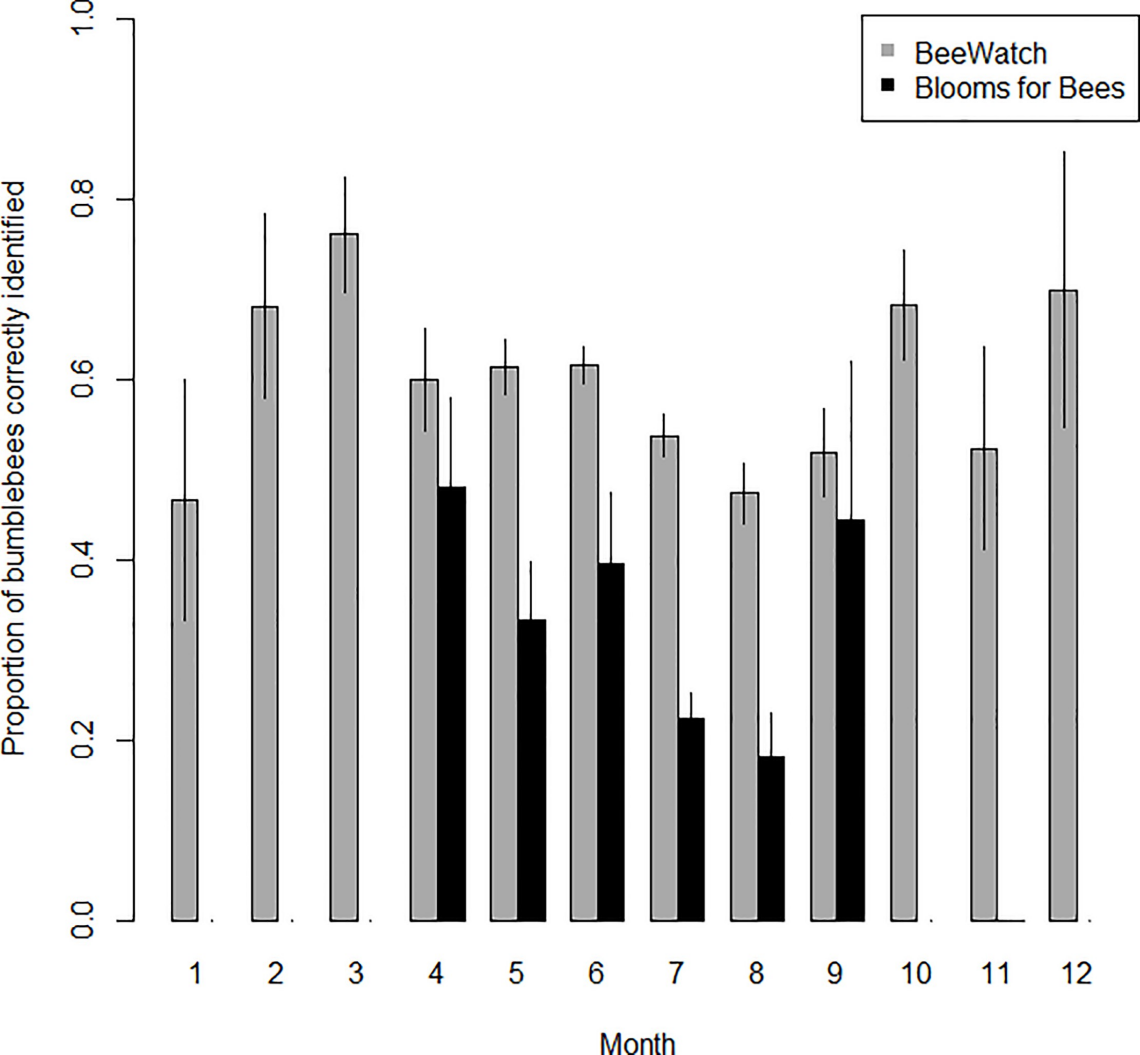
Recorder success by month, considering only each recorder’s first submitted record. Bars indicate the proportion of recorder-submitted identifications which were confirmed correct by verifiers per month and project. Error bars show ±SE.

Recorder accuracy (the proportion of expert-verified records correctly identified by recorders) varied significantly between months (Month: F_11,3481_ = 3.166, p<0.001, Fig 5). Recorder success (the proportion of recorder-submitted identifications which were confirmed correct by verifiers) did not vary significantly by month (p>0.05, Fig 6). Recorder accuracy and success rates were generally lower during the summer months in both projects (Figs 5 and 6), and BeeWatch recorders generally had higher rates of success than Blooms for Bees recorders for the majority of months. Recorder accuracy and success rates in the BeeWatch project were relatively consistent across years, although the mean success rate was significantly higher in 2011, the first year of recording (Figs 3 and 4). The project only began in the second half of 2011 so this increased success rate is likely to result from a small number of records (237) submitted by more expert recorders (as people with a strong interest in bumblebees are likely to hear about bumblebee-related projects before the general public).

Both projects had a number of ‘repeat recorders’. In Blooms for Bees 85% of recorders submitted more than one record, and 23% submitted more than 10. In BeeWatch, the figures were 46% and 6% respectively, with three recorders submitting more than 100 records. As BeeWatch recorders had several years to submit additional records we also calculated the number of repeat recorders within each full year (2012–15): 13–16% of recorders submitted more than one record, and 1–2% submitted 10 or more records, in a calendar year.

In both projects, recorder identification ability was found to improve with the number of records submitted. Where the recorders’ success for each identification was coded as 0 (wrong) or 1 (correct), within BeeWatch, recorder accuracy increased by 0.0038 (±0.00022) per record from the second submission onwards (Z = 16.75, p<0.001), dropping to 0.0031 (±0.00022) from the tenth submission onwards (Z = 13.59, p<0.001, max = 781 records) as identification ability began to plateau. Mean accuracy for each recorder’s first identification in this dataset was 31.09% (n = 3425), rising to 43.52% (n = 8077) for records from the second record upwards, and 55.02% (n = 3088) for the tenth record upwards. Within Blooms for Bees, recorders’ accuracy increased by 0.031 (±0.0024) per record from the second submission onwards (Z = 13.29, p<0.001), dropping to 0.027 (±0.0027) from the tenth record (Z = 10.26, p<0.001, max = 77 records). Mean accuracy for each recorder’s first identification in this dataset was 24.17% (n = 484), rising to 36.00% (n = 3714) for records from the second record upwards, and 44.27% (n = 1744) for the tenth record upwards.

## Discussion

This paper highlights the essential role of expert verification within citizen science projects. Here we demonstrate that, although citizen science projects can be a very effective way of gathering bumblebee data, unaided identification accuracy and success rates are generally low, especially in projects that are newly established and for species that are less frequently encountered.

The overall recorder accuracy and success rates of between 40 and 60% are lower than those reported in monitoring schemes such as the UK Ladybird Survey [19]. However, they are within the range expected, particularly for new recorders [7,19], although accuracy rates are likely to vary considerably between species groups and depending on the target audience, level of support provided, etc. The identification errors seen in both bumblebee projects were probably a result of a combination of factors, including lack of experience and the polymorphic nature of bumblebees (sexual dimorphism, multiple colour forms for some species and considerable variation for others, plus the bleaching effect of exposure to the elements) which can make them challenging to identify. Bumblebees are also often difficult to determine to species from photographs, although experts have been shown to be highly consistent in their identifications [20]. In both projects, it was not always possible to confirm a bumblebee species from the image provided. Because of this, the ‘not identifiable’ rate of 20% in Blooms for Bees and 11% in BeeWatch is likely to have contained some unconfirmable correct identifications.

As expected, recorder identification accuracy and success rates varied significantly between bumblebee species in both projects, with recorders most able to correctly identify species with distinct appearances in all castes (bumblebees have three castes: males, workers, and queens, which differ markedly in appearance for many species). Recorders achieved the highest accuracy rates for the highly distinctive species *B*. *hypnorum* (68% in Blooms for Bees and 87% in BeeWatch), and *B*. *distinguendus* (78% in BeeWatch). Recorders achieved the highest success rates for the distinct but common species *B*. *pascuorum* (72% in Blooms for Bees and 84% in BeeWatch), *B*. *lapidarius* (70% and 83% respectively), and *B*. *pratorum* (61% in Blooms for Bees). While there are other species which are morphologically similar to these species (*B*. *humilis* and *B*. *muscorum* for *B*. *pascuorum*, and *B*. *ruderarius* and *B*. *rupestris* for *B*. *lapidarius*, for example), the other species are far less abundant or widespread. This demonstrates that recorders were most successful at identifying species with distinct appearances, especially when those species were among the most common and frequently sighted.

Identification accuracy and success was very low for certain species. In the Blooms for Bees project this included three species which were never correctly identified by recorders (*B*. *jonellus*, *B*. *muscorum* and *B*. *ruderatus*) and the reporting of eight species which were not actually present in the dataset (*B*. *barbutellus*, *B*. *bohemicus*, *B*. *distinguendus*, *B*. *humilis*, *B*. *ruderarius*, *B*. *soroeensis*, *B*. *subterraneus* and *B*. *sylvarum*). Because of this, the unverified data from the Blooms for Bees project overestimated species richness by 53%. This overestimation, which results in under-recording the common species and over-recording the rare species, has also been reported by other insect monitoring projects and highlights the need for verification to avoid false positive and negative reports [7,19]. Although all but one of these species were also reported and verified in BeeWatch, the accuracy rates were lower than that of the ‘big seven’ widespread and abundant bumblebee species, which make up the vast majority of the bumblebees in gardens. This probably reflects lack of familiarity with these rarer species as well as an element of ‘wishful thinking’, as has been recorded for other citizen science surveys [7]. Using training resources to improve recorders’ basic understanding of bumblebee phenology and distributions could help reduce the incorrect reporting of species such as these.

We expected identification ability to improve over time, as a result of verification feedback and learner experience [8,30,32,43]. We found that the accuracy of bumblebee identifications varied throughout the year in both projects, but did not show an improvement across months, probably because different bumblebee species emerge and are active at different times. Accuracy was generally lowest in the summer months, and this reflects the fact that identification is most challenging at this time of year, as this is when the majority of bumblebee species are active, males which often vary in appearance from the queens and workers are also present, and the effects of wear and fading with age become apparent. The latter can result in the once distinct red-tailed, yellow banded males of *B*. *lapidarius* becoming grey-banded with an off-white tail for example, and it was noted that very few Blooms for Bees recorders were able to account for this.

We did not find a significant effect of year in data from the BeeWatch project, suggesting that the recorders did not improve en masse as a cohort. This was surprising as a five-week trial of automated natural language generated feedback was found to significantly improve recorder accuracy in the BeeWatch project [30], particularly for participants who began with below-average bumblebee identification skills. However, because citizen science recorders join projects on an ongoing basis, for a largely self-determined amount of time, this lack of project-wide improvement over time does not indicate that individuals did not learn from their involvement.

Indeed, within both projects, we found that recorders improved their identification success as they submitted more records, and that this rate decreased gradually over time as identification ability improved to a plateau (the rate of improvement was lower amongst recorders who had submitted ten or more records than amongst recorders who had submitted more than 2 records). When considered with the low frequency of occurrence of all bumblebees except the ‘big 7’ widespread and abundant species [44], this suggests that recorders quickly learn to identify the species that they encounter frequently, but may continue to struggle with less frequently encountered species or castes. Further research to assess how training resources and feedback can improve identification accuracy, learning and data quality would be beneficial.

These identification errors may also partly be a result of the photo-verification systems in place. Recorders–particularly those relatively inexperienced in bumblebee identification to species–may have been reassured by the presence of experts, and thus submitted records with identifications that they were less confident of. It is therefore possible that the provision of expert verification, which is increasingly common in recording schemes aimed at the general public, may actually decrease the quality of the identifications assigned by the recorders. The increased opportunities for mentoring and learning provided by this approach should mitigate this over time for individual recorders, as we found during this study. The finding is strongly suggestive of a changing role of recording schemes, with a movement away from simply harvesting and collating data from known recorders, towards a more inclusive teaching and mentoring role. This should have positive implications for recorders, and for the number and quality of records produced, but it makes the role of the verifier considerably more complex and time-consuming. Historically British natural history recording schemes have been led by a single volunteer–this approach is increasingly incompatible with the emerging demands of citizen science in the 21^st^ century.

The relatively low accuracy rates demonstrated in pollinator monitoring citizen science projects highlights the importance of effective training resources [25], which can have a significant effect on accuracy [26,30,32]. While both Blooms for Bees and BeeWatch provided identification resources in various formats (as outlined in the method), it is apparent that many recorders still found identification challenging. This is unsurprising, as accuracy generally varies depending on the difficulty of classifying species within a given taxon [27], with bumblebees being a notoriously challenging genus to identify, with multiple species of similar appearance, multiple castes of differing appearance for each species, and much variation within species between individuals, especially between fresh and worn individuals. This is compounded by bumblebees’ ability to sting, which reduces people’s willingness to examine individuals closely. Understanding where misidentifications frequently occurred can indicate where recorders faced the greatest challenges and therefore where training resources can be strengthened to improve recorder ability.

For example, in both projects, accuracy and success rates were low across the black and yellow banded bumblebee species, many of which were confused with each other. Most notably, in Blooms for Bees, only 5% of the records submitted as *B*. *hortorum* were confirmed as correct following verification. Of the 474 records submitted for this species, the majority of the records (70%) were reassigned to the similar-looking species *B*. *terrestris* and *B*. *lucorum* (S2 Table). This suggests that providing additional information about how the black and yellow banded bumblebee species differ from one another, and the key characteristics which define them, could improve identification accuracy for these species. This could be achieved by providing ‘compare and contrast’ images with annotations, for example in this case to highlight the midriff band present in *B*. *hortorum* but absent in *B*. *terrestris* and most *B*. *lucorum*. This may be most effective if the images are field-relevant, for example photographs or even video clips, allowing recorders to gain a better understanding and mental image of what to look for themselves.

As expected, recorder accuracy and success varied significantly between the two projects, with Blooms for Bees recorders (largely recruited from the gardening community) significantly less able to identify bumblebees than BeeWatch recorders (largely individuals with a specific interest in bumblebees). Even in its first year of operation, recorders in the BeeWatch project achieved greater mean proportions of correct bumblebee identifications than in the Blooms for Bees project. This was most likely because BeeWatch recorders had a greater familiarity with bumblebees than Blooms for Bees recorders. This is perhaps especially evident in records for *B*. *vestalis*, where BeeWatch recorders clearly recognised members of the difficult cuckoo bumblebee group (subgenus *Psithyrus*) more readily than the average Blooms for Bees recorder.

Protocol complexity may have also had an effect on recorder accuracy, as demonstrated in ladybird citizen science projects [19]. Blooms for Bees recorders were asked to record all bumblebees seen during a five-minute period, rather than selecting specimens to submit on an ad hoc basis as in BeeWatch. Blooms for Bees recorders therefore faced the challenge of photographing often large numbers of bumblebees very rapidly and possibly had less time to identify each bumblebee in the field. Consequently, this is also likely to have had an impact on the proportion of records that could not be verified because of missing or poor quality supporting photographic evidence, which was higher in the Blooms for Bees project (20% vs 11%). BeeWatch recorders were also more cautious when assigning species identifications, recording species as ‘unknown’ more often than Blooms for Bees participants (33% vs 7%), possibly because they were more conscious of the similarities between some species. This risk averse approach would have improved accuracy, as it is likely that recorders had more confidence in the species they did assign.

Although this paper only explores the accuracy of the insect data submitted to Blooms for Bees, recorders were also asked to submit details about the plants they surveyed. Identification accuracy issues also affect citizen science plant recording schemes [45–48], and in the case of ornamental garden plants misidentification can occur not only at the genus and species level, but also at the cultivar level. With over 76,000 plants available to UK gardeners [49], there is a high potential for misidentification. The verification of all data submitted by recorders is therefore essential, and especially important in studies investigating plant-animal interactions, where there is greater opportunity for error.

In order to maximise the potential for verification and minimise missing data, the importance of including supporting photographs must to be emphasised to recorders. However, this needs to be balanced with the risk of discouraging participants, who are generally less likely to submit data as more is required of them [50]. However, as potential participants’ willingness to submit data is often limited by their concerns about the quality of their data [5], providing information on the extra value added by including photographs may help assuage these fears and increase data submission rates.

It would also be worth providing detailed guidance on how to photograph bumblebees most effectively. Although out-of-focus images, or photographs from awkward angles, can often be identified by an experienced verifier who can use features such as relative size, shape, face shape, fluffiness and flower choice to inform their decision, this can slow down the verification process. Bumblebees have important identification features which are not often all visible in the same photograph (e.g. tail colour, banding pattern, face length, and hind legs), so information on which photographs are most likely to result in an identification would help improve the quality of images for verification. Additionally, tips on how to recognise photographic distortion may be useful, though these can at least be adjusted digitally to some extent during the verification process.

## Conclusion

Here we demonstrate and quantify the essential role of expert verification in bumblebee monitoring projects. The accuracy and success rates of bumblebee identification documented in this paper indicate that unverified citizen science records have the potential to severely compromise the usability of datasets and produce misleading conclusions. Without verification, there is also the risk that datasets could contaminate important national datasets such as that of the Bees, Wasps and Ants Recording Society (BWARS) and data aggregators such as the National Biodiversity Network (NBN). Given the high level of interest in citizen science pollinator monitoring schemes, our paper suggests that ensuring and demonstrating data accuracy in future projects is essential.

Expert verification improved the quality of the bumblebee citizen science datasets, and highlights the importance of the supporting photographs in order to maximise verification potential. Verification can be easily incorporated into projects, and although it can be time-consuming, it is essential for ensuring data quality. The rate of verification can vary considerably depending on the system used, the quality of the photographs submitted, the skill level of the verifier and level of interaction with recorders, although the use of automated natural language generated feedback such as that being developed as part of the BeeWatch project [51] has the potential to speed up the processing time for each individual record and thus reduce the verifiers workload.

Given the high levels of error for certain species, there is a need for high-quality training resources to support recorders. Our data analysis revealed where some of the biggest misidentification issues lie, and indicates where identification and training resources could be enhanced to improve data quality in future bumblebee monitoring projects. For citizen science using non-experts and intermediate level participants, the following is suggested:

- Provide side-by-side 'compare and contrast' images of similar species, with annotations to emphasise the key characteristic (for example, to highlight the midriff band present in *B*. *hortorum* and *B*. *jonellus* but absent in *B*. *terrestris* and *B*. *lucorum*).
- Provide resources to help recorders identify the different castes, particularly where this is crucial in separating species (for example when differentiating between male *B*. *terrestris* and *B*. *lucorum*).
- Include a range of images to emphasise the variation within species (especially for variable species such as *B*. *pascuorum* and *B*. *hypnorum*)
- Include a range of images to illustrate the effects of wear and ageing (notably for males of *B*. *lapidarius* where faded males were mostly misidentified).
- Provide information about bumblebee species phenology and distributions to help reduce the over-reporting of scarce and rare species.
- Include images of the insects that are most frequently mistaken for and recorded as bumblebees including the Honey Bee (*Apis mellifera*), the hairy footed flower bee (*Anthophora plumipes*), leafcutter bees (*Megachile* species), mason bees (*Osmia* species) and hoverflies (Diptera: Syrphidae)
- Provide detailed guidance on how to photograph bees, including how to recognise photographic distortion (such as over-exposure of bees on dark or red flowers which can result in an over-bright photo and under-exposure of bees on white flowers which can result in silhouetting and muting of body markings).

## Supporting information

S1 Protocol Blooms for Bees protocol. (DOCX)

S1 Table Data summary. Number of records submitted and verified in the two bumblebee citizen science projects, Blooms for Bees and BeeWatch. Both projects had the option to not specify a species. Neither *B*. *subterraneus* nor *B*. *terrestris/lucorum* was an option on the drop-down list of species which BeeWatch recorders had to choose from. Because both projects were aimed at recording bumblebees only, neither had an option to record sightings as members of any other group. Unknown/not identifiable records (i.e. those which could not be confirmed from the photographs supplied) may have contained some correct records.
(XLSX)

S2 Table Summarised data from Blooms for Bees. Recorder-submitted identifications are presented in columns, expert-verified identifications are presented in rows. Green cells indicate the number of recorder-submitted identifications that were verified as correct.
(XLSX)
