# Supplementary material for: Evaluating the ability of citizen scientists to identify bumblebee (Bombus) species
Source: PLoS One. 2019 Jun 24;14(6):e0218614. doi: 10.1371/journal.pone.0218614 (PMC6590798; doi:10.1371/journal.pone.0218614)
Supplement: S1 Protocol — (DOCX) [file pone.0218614.s001.docx]

**S1 Protocol**

**Blooms for Bees protocol**

**1. Register**

We need to collect some details about you and the location where you will be conducting your surveys. This will enable us to get in touch if we have queries, and help us understand the effects of location and garden management on bumblebees.

**Sign up** using your email address and name.

**Add a location** (e.g. a garden or allotment) and provide details about its type, size, location and surroundings. You can also provide details about garden characteristics, management and features if you wish.

If you would like to survey more than one garden or allotment, you can add multiple locations.

**2. Choose a flowering plant**

When you are ready to carry out a survey, you will need to select a plant to monitor. This plant needs to have at least one open flower and be located in a position which you can safely and easily watch for five minutes. You can survey any plant, including ornamental flowers, fruits, vegetables, herbs, wildflowers or ‘weeds’.

To do this:

- Start a **new survey**.
- Select your **location**.
- Record which **plant** you are surveying by typing either the common or scientific name in the plant search box, and selecting the appropriate option from the plant list.

The plant list includes many of the plants commonly grown in gardens and allotments, but if your chosen survey plant is not listed, please select **plant not listed** and type its name in the text box.

If you do not know the name of the survey plant, please select **plant unknown**, and we will try to identify it from your photograph.

You will need to estimate how many flowers are open on the plant you are surveying. Because it’s not always easy to decide what a flower is, we would like you to count or estimate the number of **floral units**.

A single floral unit is one simple flower, head, umbel or spike – these are illustrated below with examples. Essentially, a floral unit is a collection of flowers that a bumblebee could walk around; the bee would have to fly to reach another floral unit on the same plant.

If the plant is very big or has a large number of flowers, you can choose a small section to survey and report on the bumblebee visits to those flowers. In this case just record the number of floral units you are watching, rather than the number on the whole plant.

**Take a picture** of one of the plant’s open flowers and **save** the details.

| **One floral unit is:** |  | **Examples:** |
| --- | --- | --- |
| One simple flower | 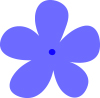 | Geranium  Nasturtium  Primrose |
| One head | 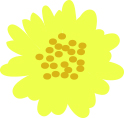 | Chive  Dahlia  Dandelion  Scabious  Sunflower |
| One umbel | 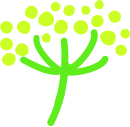 | Angelica  Cow parsley  Fennel |
| One spike or panicle | 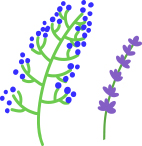 | Buddleia  Foxglove  Lavender |

**3. Begin five minute survey**

**Begin survey** and spend **five minutes** watching the chosen plant to see which bumblebees visit.

You will need to take a picture of each bumblebee that lands on the flowers you are surveying.

Only take a picture of each individual bumblebee once, so if the bumblebee moves between flowers, don’t report it again. If you have many bees in one photograph you can use the same image for multiple sightings.

Even if you don’t see any bumblebees, please submit your survey record as this will help us work out which plants are less popular with bumblebees.

**4. Photograph the bumblebees**

Each time you see a new bumblebee select the **Add New Bee** button.

If you can, **take a photo** of the bumblebee.

Try to take pictures from the side to capture key identification features. The colour of the tail is very important, as are any coloured bands on the body. It also helps if the picture shows the hind leg, as the presence or absence of a pollen basket will help us differentiate between some species.

Get close as this will allow us to see as much detail as possible. Try not to cast a shadow as this will often cause bumblebees to fly away and may affect the quality of your picture.

If a bumblebee flies away before you manage to take a picture of it, you can report the sighting without a picture.

**Tip!** You can improve iPhone pictures using a clip on lens, such as olloclip or TECHO universal lens, to allow close-up/macro shots.

**5. Identify the bumblebees**

During the survey, try to identify the bumblebee species in each picture you added by selecting a **bumblebee species** from the Bee Guide list.

You are most likely to see one of the eight common species, and probably some of the cuckoo species. The scarce species are very limited in their distributions, so check the maps in the Bee Guide to find out if they are present in your area.

Bumblebees can be tricky to identify as they are variable and often fade over time. Don’t worry if you can’t identify the bumblebee – in this case select **species unknown.** You can also choose this option to speed up the process of adding new sightings – simply go back through the records after the 5 minute survey period has finished and see if you can identify the bees!

If you want to delete a bee from the survey, you can **swipe right**.

**6. Submit data for verification**

All of your surveys and their sightings can be accessed via the **My Surveys** section of the app. When you have finished your survey and you are happy with your IDs simply **submit** the survey.

In **My Surveys** you can also delete surveys (by **swiping right**) and find out if your records have been verified. Once they have, you will be able to see if you managed to identify the bumblebee species correctly.

We hope to check and verify your records within a couple of weeks, but if we receive a large number records this may take a little longer.

If your photograph is clear enough for a definite identification, and you have given us permission, we will share your record with the National Biodiversity Network (<https://data.nbn.org.uk/>) which will contribute to long-term records, and help scientists and politicians understand and track bumblebee distributions.
